# How and why does mode of birth affect processes for routine data collection and use? A qualitative study in Bangladesh and Tanzania

**DOI:** 10.1371/journal.pgph.0003808

**Published:** 2024-12-31

**Authors:** Harriet Ruysen, Tamanna Majid, Donat Shamba, Shema Mhajabin, Jacqueline Minja, Ahmed E. Rahman, Titus Ngopi, Mary Ramesh, Shams El Arifeen, Rosie Steege, Janet Seeley, Joy E. Lawn, Louise T. Day, EN-BIRTH-2 Study Group

**Affiliations:** 1 Department of Infectious Disease Epidemiology and International Health, London School of Hygiene & Tropical Medicine, London, United Kingdom; 2 Maternal and Child Health division, icddr,b, Dhaka, Bangladesh; 3 Department of Health System, Impact Evaluation and Policy, Ifakara Health Institute (IHI), Dar es Salaam, Tanzania; 4 Department of International Public Health, Liverpool School of Tropical Medicine, Liverpool, United Kingdom; PLOS: Public Library of Science, UNITED STATES OF AMERICA

## Abstract

The World Health Organization recognises Routine Health Information System (RHIS) data as integral to data-driven health systems; needed to improve intrapartum outcomes for maternal and newborn health worldwide. However, research in Bangladesh and Tanzania suggests that mode of birth affects register data accuracy, but little is known about why. To address this gap, we undertook qualitative research in these two public-sector health systems. We conducted 44 in-depth interviews in Bangladesh (Sept-Dec 2020) and 35 in Tanzania (Feb-April 2023). Participants included health and data professionals, managers, and leaders from sub-national and national levels. Thematic analysis was undertaken with inductive and deductive coding. Emerging themes were compared/organised using determinants outlined in the Performance of Routine Information System Management (PRISM) framework. Mode of birth affected RHIS data as one part in a multidimensional system; having a caesarean changed the location of birth, availability of health professionals, and the care pathway, impacting data flow and documentation processes at facility-level. Standardised registers were available in the labour wards, but not in all operating theatres. Health professionals in both countries described feeling overwhelmed by duplicative data tasks and competing clinical care responsibilities, especially in labour wards with low staffing ratios. Health professionals perceived electronic data systems to increase duplication (for all modes of birth), along with other organisational factors. In conclusion, mode of birth influenced processes for routine data collection and use because it affected where, what, when, and by whom data were recorded. We found challenges for capturing register data, leading to potential data gaps, especially for caesarean births. Our findings suggest a broader lens is needed to improve the systems, collection, and use of individual-level data for aggregation, not just registers. Co-design of RHIS processes and tools could rationalise the data burden and increase availability and quality of perinatal data for use.

## Introduction

Increasing the availability and use of routine health information system (RHIS) data is prioritised by the World Health Organization (WHO) as an integral component of health system strengthening [1, 2]. Four in every five births worldwide are now in health facilities, but progress for the approximately 4.5 million potentially preventable maternal and newborn deaths and stillbirths every year has slowed [3, 4]. There is wide recognition that high-quality respectful care is required to improve outcomes, experience of care, and equity [5, 6]. However, responsive data-driven health systems are required to track progress and inform the necessary policy, management, and accountability mechanisms [4, 7], meaning better availability of timely, accurate RHIS data to inform improvements at the point of care, as well as for management at facility, district, national and global levels [8–10].

A series of studies have assessed the performance of perinatal health indicators for RHIS measurement [11–15]; and some evidence suggests that RHIS data have the potential to accurately capture information on complex clinical interventions [13, 15–17]. Research in Bangladesh, Nepal, and Tanzania (the EN-BIRTH study) compared direct observation of perinatal care with routine labour and delivery (L&D) ward register data [18]. Register design and completion in Bangladesh and Tanzania lacked standardisation and register-recording accuracy for some data elements varied by mode of birth [14, 19]. For example, the recording of prophylactic uterotonic administration, neonatal resuscitation, and chlorhexidine cord cleansing were more accurate for vaginal births than caesarean sections [20–22]. Conversely, recording of early initiation of breastfeeding was less accurate [23], but it is unclear why. Such variations in data recording could have measurement implications for the rapidly increasing number of caesarean births in Tanzania and Bangladesh, as well as globally (estimated to be 30% by 2030) [24]. However, there is limited literature regarding how mode of birth affects RHIS data processes in these settings, or whether this contributes to data quality for use.

Health professionals in Bangladesh and Tanzania complete various RHIS tasks (including data collection) as part of care provision, facility management, practice development, and accountability processes. Individual-level client data are captured in clinical notes/records to inform case management and can be used for quality improvement [18]. Paper-based registers in health facility wards are used to collect a subset of data elements regarding birth outcomes, service provision, and length of stay. These RHIS data are then aggregated to track health indicators (including caesarean section rates) for use at sub-national, national, or global levels as part of the RHIS [25, 26], so potential differences in registers and data flow warrant further investigation.

Our study therefore included two objectives. First, to map data flow and content for pre-printed nationally standardised (paper-based) registers (in labour wards and operation theatres (OT)) for Bangladesh and Tanzania. Second, to explore how and why mode (vaginal or caesarean) and location of birth (labour ward/OT) affects routine data collection and use in Bangladesh and Tanzania.

## Materials and methods

### Study setting

We focused on public-sector services; Bangladesh and Tanzania have national strategies to improve maternal and newborn health (MNH) data [27, 28]. Both countries have well-established RHIS, including the national use of District Health Information Software-2 (DHIS-2) for collecting, managing, and using electronic RHIS data [29]. In Bangladesh, public-sector MNH services are provided by the Director-General Health Services (DGHS) and the Director-General Family Planning (DGFP) [30]. In Tanzania, public MNH services are governed by the Ministry of Health and Social Welfare [31].

We embedded this qualitative research within a mixed methods study that developed and tested tools to improve the availability, quality, and use of newborn and stillbirth indicators in RHIS; the **E**very **N**ewborn-**M**easurement **I**mprovement for **N**ewborn & Stillbirth **I**ndicators (EN-MINI)

Tools [32]. Although the EN-MINI tool focus was more general, we were able to undertake our qualitative data collection in the same public health facilities, plus national and regional offices at the same time.

### Design

We undertook this phenomenological qualitative research within a constructivist paradigm using in-depth interviews in Bangladesh (1^st^ Sept-21^st^ Dec 2020) and Tanzania (21^st^ Feb- 30^th^ April 2023). The semi-structured interview guide was adapted from the ’Assessing Barriers to Data Demand and Use in the Health Sector’ tool [33] (S1 Text).

### Mapping of data elements in registers

We mapped the data elements of the standardised paper-based L&D registers used at participating health facilities. At the time of data collection, standardised OT registers were not being used for caesarean births and were therefore excluded from mapping.

### Participants, sampling, and recruitment

**Fig 1:**
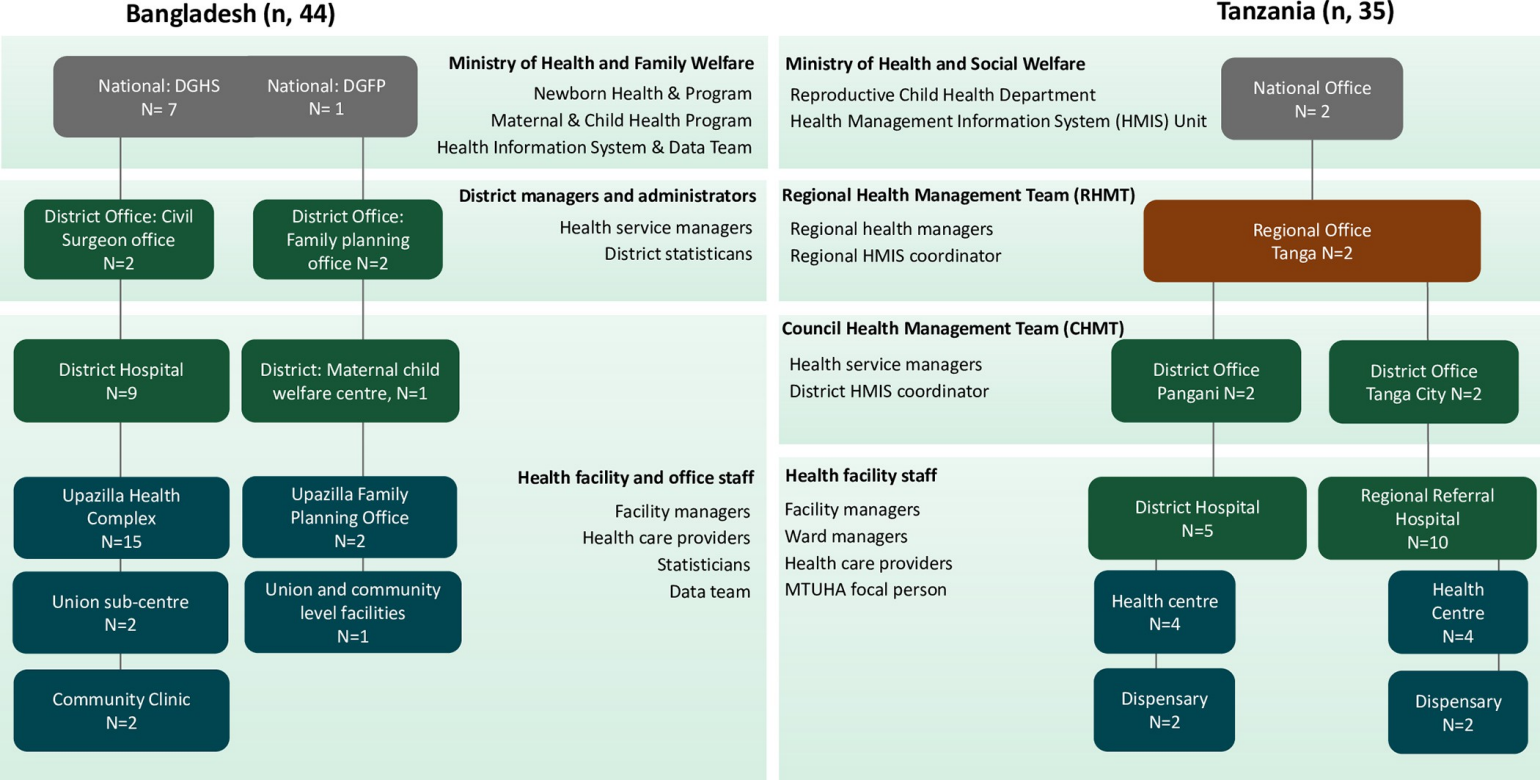
Participants by level of the health system. Fig 1 shows 2 pyramids side by side, one to represent Bangladesh and one to represent Tanzania. The base of the pyramid represents the different health facilities and offices that participants were recruited from at community level (colour coded in blue). The next layer represents the district-level facilities and offices, these are dark green. For Tanzania only, the regional level office is in dark orange. At the top of the pyramid, national-level offices are grey.

Participants were from facility, sub-national, and national levels of the healthcare systems in Bangladesh and Tanzania. Participants were purposively selected to include a diverse range of roles, levels of care, and responsibility at different levels of the health system health (including health and data professionals, managers/leaders, and policymakers) (Fig 1).

In Bangladesh, district and facility-level participants were selected from both Directorates (DGFP and DGHS) in Kushtia district. The sampling frame included district-level offices (2), Upazilla-level office (1), district hospitals (2), Upazilla health complexes (5), Union sub-centres (3), community clinics (2). In addition to national-level offices (2) for DGFP and DGHS.

In Tanzania, district and facility-level participants were selected from two districts in Tanga Region: the sampling frame included district-level offices (2), hospitals (2), health centres (4), and dispensaries (4). As well as the regional and national offices (2).

### Data collection and management

Experienced qualitative researchers (TM, DS, SM) interviewed participants in their preferred language (Bengali, Swahili, English). Interviews took place in a private and mutually convenient location of the participant’s choice; they lasted 45–75 minutes. Seventy-seven participants consented to audio-recorded interviews, and two (1 for each country) consented to written documentation instead. Further information concerning non-verbal communication, and the general tone and atmosphere were documented in an interview summary. Research assistants transcribed and translated the interviews into English, and transcripts were checked by the interviewing researchers.

The research team engaged in regular reflexive meetings at all stages of the study, considering our positionality, the conceptualisation of preliminary themes, and the ongoing refinement of the interview guide and recruitment strategy (S2 Text). Data collection and analysis were concurrent, allowing emerging themes to be investigated in later interviews. The sample size was met when no new information emerged about our research questions.

Transcripts were de-identified, and aggregated descriptors were used. The participant’s demographic characteristics are stored separately from the transcript. All records are managed and stored according to agreed data protection guidelines.

### Ethical considerations

This study was granted ethical approval by the Bangladesh Research Review Committee (icddr,b), Ifakara Health Institute, the National Institute for Medical Research (Tanzania), and the London School of Hygiene & Tropical Medicine (United Kingdom) (S1 Table). Information regarding the study objectives, process, and intended data use was shared with potential participants in line with approved participant information and written consent forms (S3 Text). Participants were reassured of their right to withdraw consent without consequence. We followed the Standards for Reporting Qualitative Research recommendations (S1 Checklist).

#### Inclusivity in global research

Additional information regarding the ethical, cultural, and scientific considerations specific to inclusivity in global research is included in S4 Text.

### Qualitative data analysis

**Fig 2:**
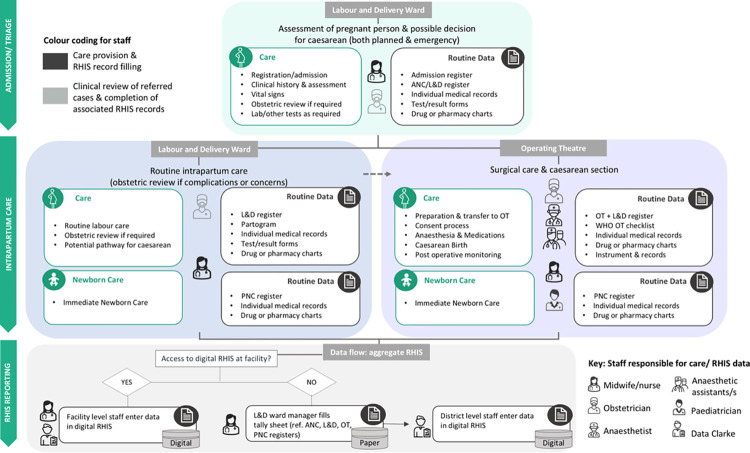
Data flow by place, people, and care pathway comparing vaginal births on labour and delivery ward and caesarean sections in operating theatre. This flow chat maps the care and data journey from admission to birth for vaginal and caesarean births. Care activities are shown in green and linked to a pregnant person icon, data activities are in grey and linked to a chart icon. The key staff responsible are also mapped using icons to represent different professionals.

HR, DS, and TM undertook thematic analysis following the six phases developed by Braun and Clarke [34, 35]: familiarisation with data, initial line-by-line coding, theme generation, review, definition and naming of themes, and finally, synthesis and conclusions. Supported by NVivo software [36], both inductive and deductive analytical approaches were used. Initial open coding informed the development of the coding framework and the data flow (Fig 2). Next, codes were organised using categories from the Performance of Routine Information System Management (PRISM) framework [37]. PRISM includes three main determinants of RHIS performance: a) Technical factors relating to the tools and technologies used within RHIS’ for data collection, processing, and analysis. b) Organisational factors relating to the policies, leadership, and culture underpinning the RHIS structure and environment. c) Behavioural factors relating to individual perceptions and experiences that interact with how RHIS users support and engage with the RHIS [38]. Consensus was reached through regular analysis discussions.

## Results

We interviewed 79 participants (Bangladesh n,44; Tanzania n,35) (Table 1). Half (n,43) had ten or more years of experience working in the public health sector. Most (n,50) were aged 35 or older, 38 identified as male, and 41 as female.

**Table 1:** Demographic characteristics of participants.

	Bangladesh (n,44)	Tanzania (n,35)	Total (n,79)
**Gender**			
Male	21	17	38
Female	23	18	41
Gender-expansive	0	0	0
**Age**			
18 to 24	1	0	1
25 to 34	14	14	28
35 to 44	13	14	27
45 to 54	12	4	16
55 and over	4	3	7
**Highest qualification**			
Master’s degree	16	4	20
Batchelors Degree	11	9	20
Diploma/certificate	16	21	37
Completed secondary school	1	1	2
**Role/Cadre**			
National-level leader	8	2	10
Regional-level manager	0	2	2
District-level manager/ coordinator	3	2	5
Data focal person	0	4	4
Statistician	4	0	4
Facility manager	0	8	8
Ward manager	9	6	15
Doctor	6	1	7
Nurses/midwife	5	8	13
Other health professional	9	2	11
**Years in current post**			
0 to 4	27	16	43
5 to 9	9	17	26
10 to 29	7	2	9
30 or more	1	0	1
**Overall years in public health service**			
0 to 4	7	4	11
5 to 9	9	16	25
10 to 29	26	13	39
30 or more	2	2	4

We found that mode of birth affected, where, what, and when data were recorded, and by whom. The findings are presented in sections starting with the those most closely linked to mode of birth and concluding with issues that transcended mode of birth:

- Data flow and registers differed by mode of birth with potential RHIS data gaps for caesareans.

Themes from the qualitative data analysis:

- Digitisation worsened the duplication for fragmented RHIS and increased the data burden, especially for over-stretched L&D ward staff.
- Health professionals were overwhelmed by duplicative RHIS tasks which competed with providing care, especially in L&D wards where there were lower staff ratios.
- Organisational factors were determined by facility or higher-level policies, resourcing, and culture (rather than mode of birth).

### Data flow and registers differed by mode of birth with potential RHIS data gaps for caesareans (technical factors)

In Bangladesh and Tanzania, having a caesarean changed the location of birth (L&D ward/OT), availability of health professionals, and care pathway, impacting the data flow (Fig 2). In all facilities, participants explained that admission and triage were initiated in the L&D ward. Women progressing to a vaginal birth remained in the L&D ward, and care was usually provided by a designated nurse or midwife. Those requiring a caesarean were transferred to the OT, with referral to a different facility if surgical services were not available.

In Bangladesh, both the Directorate for Health Services (DGHS) and Directorate for Family Planning (DGFP) use the Emergency Obstetric and Neonatal Care (EmONC) register (rather than an OT register) for all modes of birth. The EmONC registers are similar however, DGFP included three data elements not collected in the DGHS (last monthly period, ’reason for a caesarean’, ’type of anaesthesia’) (S2 Table). The EmONC registers were kept in the L&D wards (rather than in the OTs). Some facilities occasionally used an older version of the delivery and OT registers during stockouts of the current EmONC register. Co-ordination for L&D and OT data was challenging and health professionals generally prioritised completing medical records during the admission, leaving the register filling until discharge.

‘*We don’t write all their information when they get admitted. Suppose a patient came with labour pain, in this case, until the delivery is done, we don’t fill out the [register] cells. That remains blank. When everything is done and she will be discharged, then we fill out the cells.’*

*Midwife*, *District Hospital*, *BD*

In Tanzania, the national RHIS, Mfumo wa Taarifa za Uendeshaji Huduma za Afya (MTUHA), is a series of registers designed for primary data collection (’MTUHA 12’ for L&D ward). Standardised OT registers are not used and processes for recording caesarean birth data in the register varied between facilities. In five facilities in Tanzania, a L&D ward midwife would move to the OT to provide immediate newborn care and collect/complete the L&D register on their return. Many facilities had informal (hand-written) registers, referred to as ’counter’ books, for use in the OT. Participants reported using this information to complete and cross-check documentation (e.g. the number of caesarean births) in the L&D ward register. However, limitations were mentioned: a regional manager said, *’If you use the counter book*, *you won’t collect all the data because there is not enough space to be able to fill what is required*.*’* Two participants (district and facility-level) reported concerns that data regarding caesareans were miscounted and there were noted recording gaps regarding care provision data for caesarean births, for example: *’…details for anaesthesia such as complications or how much the patient was bleeding*, *we don’t have a place to record that information*.*’ Nurse in-charge of OT (District hospital)*, *TZ*.

Neither Bangladesh nor Tanzania had a standardised process for managing data flow for caesareans leading to potential RHIS data gaps for caesarean births.

### Digitisation worsened the duplication for fragmented RHIS and increased the data burden, especially for overstretched staff on L&D wards (technical factors)

In Bangladesh and Tanzania, duplicate RHIS tasks required health professionals to enter the same information in multiple paper-based and electronic systems (e.g., individual medical records, different registers, forms, and tally sheets, or databases). This was highlighted by staff from all facilities in Bangladesh and 8/10 facilities in Tanzania. L&D staff (and staff in lower-level facilities) were more impacted by duplicative processes than those working in OT, for example:

*’If a mother enters the labour ward, we have a register with 47 columns where we enter… there’s information about ANC and admission. There’s some about PNC as well. We also have to make online entries for each admission in the labour ward. ANC doesn’t have online entries, but labour ward does.’*

*Midwife (District hospital)*, *BD*

Implementation of electronic RHIS systems differed between and within countries, including processes for where and who digitised RHIS data. Many staff lamented that the introduction of eRHIS which required them to complete both paper-based and eRHIS records with duplicate information, and created a double recording burden.

’*We usually have double work, which means we have to get time to record data on paper and then record it in the system. This increases the workload; when you are busy, it reaches a point where you will not record in either.’*

*Nurse*, *recovery (District hospital)*, *TZ*

In Bangladesh, both directorates were progressing towards scaling up DHIS-2 within all facilities. As with the paper-based registers, data for vaginal and caesarean births were aggregated together in the same DHIS-2 form but poor alignment between eRHIS forms and the registers was noted by a district manager as challenging.

Tanzania also used DHIS-2 software. In addition, several facilities used supplementary RHIS software (AFYA CARE, Kanzi Data, and GoT-HoMIS) and highlighted the imperative to integrate systems and remove redundancy.

’*I think that if GoT-HoMIS is integrated it will be much better than entering data in GoT-HoMIS, and in the paperwork, at the end of the month, you enter the report manually, then enter it in the system. If they are integrated, after completing to enter data in GoT-HoMIS at the end of the month you will just generate the data, such as exporting in DHIS2. You get rid of the paperwork.’*

*Regional manager*, *TZ*

These technical factors contributed to the duplicative data burden and fragmentation that electronic systems should help solve. Especially given that health professionals from the L&D ward were responsible for a large variety of different registers and forms. Many extended well beyond the scope of intrapartum care and remined largely paper-based, despite implementation of electronic systems (DHIS-2):

’*There are a lot [of registers]! For example, childcare, general patient care, mother and newborn baby care, inventory, and combined register. In addition to that, there is also a pill supply register and two different registers for injections. There is also an IUD [intrauterine device] register with three different sections. I have to fill out those as well.*

*Senior staff nurse (District hospital)*, *BD*

There were also noted design challenges for registers. Despite the plethora of existing registers, some participants requested a separate register for newborns, especially in Bangladesh, where the register included many free text boxes. Six participants from Bangladesh (none from Tanzania) complained that the cells were too small. Three health professionals from Bangladesh also highlighted that the small cell size impeded the accurate medication recording.

Each data entry (whether paper-based, or at the point of digitisation) represents an opportunity for error and an associated requirement for quality-checking processes. Quality checks are necessary, but participants explained that this was labour-intensive and involved multiple staff at different health system levels:

’*It means that once the report is written, the final monthly report must be reviewed by two or three people from the ward before we submit it.’*

Assistant nurse (District hospital), TZ

’*Then our in-charge and seniors come and find our mistakes*. *And we are accountable to these mistakes… they are checking the register book and also cross-checking the registered data to check whether we have given them the authentic information*.*’*

Community health care provider, BD

’*[During a spot check] I’ll open a certain Register*, *open Tally sheet*, *open a summary form and open DHIS2*. *Is the information entered in those areas the same*? *That is mostly what is checked*. *Number two*, *are the information entered realistic*? *This spot-check should be done daily*, *but sometimes because of people being busy*, *it becomes hard to do daily*.*’*

*Nurse In-charge (Dispensary)*, *TZ*

This top-down quality process had some unintended consequences which are detailed in the below section regarding organisational factors. Our findings also highlight the missed opportunities for better integration of mechanisms within electronic systems to monitor and support data quality.

### Health professionals were overwhelmed by duplicative RHIS tasks which competed with providing care (behavioural factors)

Participants’ testimonies consistently focused on insufficient human resourcing more than other dimensions of RHIS performance. The sense of being overwhelmed permeated accounts from healthcare professionals in both countries, for example:

’*We have to maintain maternal care, and we also have to look after the baby as well. So, there is often a rush of patients, so we stay busy. We have to clean up the mess after the delivery and also take care of the baby. I feel disoriented in those moments.’*

*Community Health Care Provider (Community clinic)*, *BD*

This tension was exacerbated in labour wards, during night duties, or in lower-level facilities where a single health professional was managing multiple responsibilities. Although this challenge was partially associated with mode of birth (e.g. worse in L&D wards), many of the potential causes and solutions are higher up in the health system.

The ratio of staff was higher in OTs, increasing the availability of staff for care provision and RHIS tasks. Participants explained that in the OT, responsibilities for completing individual clinical records and informal OT registers were shared between nurses/midwives, doctors, and other supporting staff.

’*In labour [ward], nurses are the ones who fill in the data; in theatre, there is a combination of doctors and nurses. This can cause other data to be missed in our MTUHA.’*

*Nurse*, *maternity (District hospital)*, *TZ*

This contrasted with task allocation in the L&D ward, where a single staff member was usually responsible for multiple RHIS processes (e.g. completion of registers, medical records, reporting) while simultaneously providing care (often to multiple people, despite the importance of one-to-one care during labour) (Fig 2).

’*Sometimes it’s difficult for us to maintain so many registers. Also, the person making the entries finds it really difficult to do it due to the pressure from so many patients and so much work. Sometimes, it’s even hard to complete the reports in time.’*

Midwife (District hospital), BD

’*After the end of the month*, *between the 1st and 5th date*, *we are supposed to draft a report*. *We have one report*. *We have a report for the theatre register and another from the labour ward*, *and we merge them into one report*. *…the report from labour shows the ones who had surgery or a normal delivery*. *So*, *in labour [ward]*, *one book has information from theatre*.*’*

*Nurse*, *OT (District hospital)*, *TZ*

The burden was also intensified by monthly reporting cycles because staff were divided between conflicting responsibilities for care provision and data reporting.

’*…due to the scarcity of staff, sometimes we forget to enter some information, which becomes a big challenge when closing the monthly report. You might find yourself alone in the ward, or sometimes a mother gives birth quickly, and you hastily write her file and put it aside. Then you go, assist another mother, write her report, and put it aside. When it comes time to enter the data, you might forget to input the information for the first mother, and her data might get lost. A client may deliver at night, but her information can’t be documented until morning.’*

*Assistant nurse (District hospital)*, *TZ*

Staffing shortages impacted data use as well as data collection. Most participants described RHIS data as important and useful. This was embodied by accounts of staying late to complete RHIS duties (n,8) or using personal resources to fulfil tasks (e.g., phone credit). However, most frontline workers conceptualised the importance of data use in terms of their clinical case management, handover, procurement of medical supplies, and accountability (rather than to inform service management, quality of care improvements, changes in population health status etc).

’*If you can provide accurate information, even our workload can be visible. For example, if you say you’re busy, but you’ve performed 20 operations, it won’t convince anyone that you have a heavy workload. However, if you say you’re busy and you’ve conducted nearly 200 operations in a month, then it truly appears that there is a significant workload. This can persuade others to consider increasing manpower and providing more medical equipment and medications.’*

*Doctor (District hospital)*, *TZ*

Health professionals expressed the need to prove what they had done, especially with the high volume of cases. In Bangladesh this may be exacerbated by a hierarchical medical model, as explained by a midwife:

’*I have to keep documents of what I am doing. If I said through my mouth that I did ten deliveries in the last month, would you believe me? Would the government believe me? I do my job, and there is a document showing that. When you see that, you will understand.’*

*Midwife*, *labour ward (Upazilla)*, *BD*

The testimonies highlight a conflict between needing data for accountability, to create evidence for staffing increases, and for protection (e.g. MPDSR, complaints).

### Organisational factors were determined by facility or higher-level policies, resourcing, and culture (rather than mode of birth)

Factors such as governance, training, and supervision were similar for vaginal and caesarean births in both countries. This was partly because data for caesarean births were collected and aggregated in the same L&D ward registers as vaginal births. For example, many participants prioritised the completeness and timeliness of register filling over other dimensions of RHIS data quality and use.

’*Sometimes it’s just the attitude of people because they fill in just to avoid being told they haven’t filled in. So, they fill in just to appear as if they’ve done the job…. Sometimes, they are not filled in accurately, but they are filled in just on time’.*

Doctor (District hospital), TZ

’*Our supervisor*, *sir*, *handles this with his own hands*, *and he tells us how to do it in a proper way…*. *They tell us to fill up all the cells in the register and they check in to this*. *They also cross-check the total summary and the report*.*’*

*Midwife (Upazilla)*, *BD*

This was reinforced by data reporting structures and supervision approaches. Supervision was top-down with a strong focus on quality monitoring, especially the completeness of registers and tally sheets (rather than localised data use), which negatively impacted on participants sense of data ownership and wellbeing. Participants wanted more positive encouragement.

’*They come and then look at all the registers. They check if we are doing our thing right… They used to come a lot. They used to catch our mistakes often and made us listen to their harsh words because of the mistakes we made.’*

*Family Welfare Visitor (assistant nurse*, *Maternal and Child Welfare Centre)*, *BD*

Data use varied by role, level of responsibility and training, but frontline health professionals were also overstretched with practical limitations on their capacity to take on additional tasks. Programme and facility managers were more likely to discuss data use for public health or higher-level service planning. In Bangladesh, the strong hierarchical structures meant that lower-level and frontline staff were not empowered for local data use. This was generally perceived as being the responsibility of more senior colleagues.

’*Our job is to submit the report. We don’t know what further is done with it. We provide a monthly report to the statistician, and after the data entry, he sends it where needed. We don’t have any other authority over these data.’*

*Midwife*, *labour ward (District hospital)*, *BD*

This was reinforced by participant accounts around in-service training. Increasing access to training was valued by many, but health professionals generally expressed interest in training on data collection and register filling (rather than interpretation and use). These organisational factors were determined by facility or higher-level policies, resourcing, and culture (rather than mode of birth).

## Discussion

To our knowledge, this is the first study to investigate how mode of birth effects routine data collection in Bangladesh and Tanzania. We found that having a caesarean changed the location of birth (L&D ward/OT), availability of health professionals, and care pathway, impacting the data flow (Fig 2). Consequently, mode of birth affected, where, what, and when data were recorded, and by whom. Despite differences in national contexts and health system designs, four key themes cross-cut in Bangladesh and Tanzania: 1) Data flow and register use for caesarean births were inconsistent, leading to potential RHIS data gaps. 2) Digitisation worsened the duplication for fragmented RHIS and increased the data burden, affecting over-stretched L&D ward staff most. 3) Health professionals were overwhelmed by duplicative RHIS tasks which competed with providing care, especially for vaginal births in L&D wards where there were lower staff ratios. 4) Factors such as motivation, data use for decision-making, competence, and confidence with RHIS tasks were determined by the participant’s professional role, local or higher-level policies, resourcing, and culture (rather than mode/location of birth).

Our findings show how different data systems in OT and L&D wards contribute to the variation in data quality reported in Bangladesh and Tanzania. In L&D wards, standardised printed registers were available, but not in all OTs. Consequently, data flow for caesarean births varied between facilities. This contributed to potential data gaps for caesarean births and is consistent with survey findings from 24 Lower- and Middle- Income Countries (LMIC), where only 18 reported that the number of caesareans are aggregated in their RHIS summary form [15]. Inequitable provision of caesareans in Bangladesh, Tanzania, and worldwide means that caesareans are disproportionately high for richer, more educated, urban women and disproportionately low for poorer, less educated, rural women [39, 40]. *Too much too soon or too little too late*; a serious public health concern, especially in low-resourced health systems [41, 42]. Despite a clear need for timely data to track and respond to these challenges, our study showed that opportunities for RHIS data were not being fully realised.

The electronic transition for RHIS and medical records could facilitate more efficient collection and use of data (aggregate and individual-level) than is feasible in paper-based systems [2]. However, we found that electronic systems added to the RHIS burden, especially in L&D wards with high staff workloads. Interoperability issues and lack of standardisation further complicated data management, echoing challenges documented in a range of high- low- and middle-income contexts [43–49]. We identified the risk for losing caesarean birth data in the gaps between different OT and L&D ward systems, but the risk does not end here. Linked, yet individual, needs for the newborn add a further dimension of complexity and compound the necessity for genuine interoperability that allows easy aggregation of data from different locations and sources [49]. However, evidence from sub-Saharan Africa suggests that resource limitations have led to a plethora of short-term outcome-orientated digitization endeavours for RHIS across the region that fail to address these challenges. Dehnavieh et al. [46] also agree that the evolution of RHIS in many LMICs has been fragmented. This has been to the detriment of more holistic approaches that meet the reality of complex healthcare systems, especially for those expected to input the data at source (usually health professionals who are simultaneously responsible for care provision) [50].

Scale-up of interoperable systems that include individual-level data could facilitate measurement of caesarean care coverage and provision (e.g. quality and timing) in Bangladesh and Tanzania. WHO recommends implementing Robson classification to inform efforts to reduce unnecessary caesareans [4, 51]. Robson classification uses six obstetric characteristics (parity, number of foetuses, previous caesarean, onset of labour, gestational age, and foetal presentation) to categorise women and compare caesarean rates over time and between facilities [51]. These variables are usually collected in clinical records and require individual-level data. However, inequitable scale-up of individual-level data systems means most of the studies using Robson classification were undertaken in Europe and North/Latin America [52–54]; LMICs are underrepresented, especially in sub-Saharan Africa. However, we need better data on caesarean provision to understand and address these disparities, and to ensure timely access for those in need (Table 2). Detailed mapping of current RHIS data elements compared with existing quality of care frameworks for caesarean births will be a helpful first step in Bangladesh and Tanzania. This may also inform the prioritisation of feasible RHIS measures for monitoring the provision and quality of caesarean births in real time at facility, district, national and global levels (and over-time).

**Table 2:** Next steps.

Programmatic opportunities now	Research gaps
Use a systematic process e.g. EN-MINI Tools to: • Review indictors and reduce number. • Identify duplication between paper-based & digital RHIS systems for removal. • Promote supportive supervision as an opportunity for local data ownership and use	• Co-design of RHIS tools and process (inc. registers in L&D and OT) with healthcare professionals and other stakeholders to track core indicators. • Understand and improve measurement of experiences of birthing people and their companions (including potential effects of RHIS on experience of care). • Non-public sector (inc. private, non-governmental etc) need to be included in both the research and policy making, especially for issues such as caesarean section, which is much higher in the private sector. • Develop and test a parsimonious list of quality-of-care indicators for caesarean births (e.g. timing etc.)

That said, health professionals were burdened by duplicative RHIS tasks which competed with providing care. Lower ratios of L&D ward staff meant this was exacerbated for vaginal births, however, these challenges were cross-cutting and heavily influenced by higher-level policies and financing. However, the documentation burden is increasing in a paradoxical scenario where resourcing is not keeping pace with the rapidly growing demand for data. Health professionals from the North America, Europe, Asia, and Australia spend roughly one-third of their working day on RHIS tasks [48, 55]. Research in Bangladesh and Tanzania also shows that documenting MNH data is a considerable time burden [19, 56, 57]. Consistent with LMIC studies reviewed by Molenaar et al. [49], participants described staying ’late’ to complete documentation, rather than compromise on care provision. An evidence review with data from mainly high-income contexts linked the pressure to document without sufficient time allocation with poor job satisfaction and increased levels of burnout [58]. Nine of the included studies were from North America and one from sub-Saharan Africa, none were from Asia, and none had an explicit focus on perinatal care. Subsequently, Derecho et al. [59] focused their review on LMICs (11 African, 10 Asian); but only three explored time limitations as a potential factor impacting on the use of electronic systems and none considered burnout as a potential challenge. This existing research also focuses more strongly on physician’s perspectives, suggesting a more diverse range of stakeholder voices is required; especially from midwives and nurses who have the major responsibility for data capture [19] and from service users (whose experience is not well explored in low-resource settings). The evidence-base would also benefit from wider representation of private and non-public perinatal care providers.

The tension between the human experience and achieving high data quality is nuanced and not easily represented in frameworks such as PRISM [38]. PRISM conceptualises the key determinants of RHIS performance in terms of technical, organisational, and behavioural factors. These were useful for exploring potential opportunities to optimise RHIS performance but had less utility for understanding how health professionals navigated competing priorities between care provision and RHIS tasks. Especially given the ever-intensifying focus on RHIS data for individual-level management, and population accountability mechanisms. In our study, hard-work was perceived as invisible, and data were often valued as a way of legitimising and proving what had been done. In a qualitative review of MNH reporting in LMICs, Molenaar et al. [49] characterise this phenomenon as ‘data for protection’. This kind of emergent behaviour is characteristic of malfunctioning systems where technical or organisational RHIS factors are absent or misaligned. Another example of this in our study was that register completeness was valued highly (rather than accuracy) within supervision cycles and consequently, by those entering the data. Similarly, ethnographic research from Uganda [60], and quantitative PRISM assessment results from 12 African and Asian countries, show that data completeness is often valued above other dimensions of RHIS data quality and use [14, 32, 61].

Our findings build on the wider literature to show that the experiences of birthing people and their care providers should be at the heart of RHIS processes and tool design, including approaches to supervision (to ensure it is genuinely supportive). Humanising data systems to create an enabling environment for health professionals is critical given the projected workforce shortages and the association between burnout, time pressure, high workloads, and poor organisational support [62]. Engagement with stakeholders (service-users, health professionals, managers, and policymakers) in the design and implementation of RHIS (electronic and paper-based) could help optimise systems and identify/improve dysfunctional processes [46]. For example, in Ghana, data quality and engagement from facility staff were improved by streamlining from 27 different registers to one [63]. There is still a paucity of research exploring how the potential risks of undermining care provision in different settings can be balanced and mitigated to maximise the benefits of achieving data-driven health systems.

### Strengths and limitations

The participation of 79 health professionals, managers, and policymakers from public health services (and 22 different facilities) in Bangladesh and Tanzania ensured a diversity of experiences and perspectives were represented in the data. The analysis revealed that the effect of mode of birth on RHIS data processes was most evident at the point of data collection (within facilities). The themes were also powerfully coherent between Bangladesh and Tanzania, despite differences in national context and health system structure. Our qualitative research was undertaken as part of a study to develop and test the EN-MINI tools. Quantitative data (using the EN-MINI tools) were therefore collected from the same facilities and offices at the same time; they showed strong performance in data quality and use at district-level, with opportunities for focused action in health facilities [32].

As a qualitative study, we intended to contribute to a deeper understanding of how and why mode of birth effects routine data within the context of Bangladesh and Tanzania, rather than produce broadly generalisable findings. Bangladesh and Tanzania enjoy high government engagement in improving RHIS data, and both countries participated in the previous EN-BIRTH studies regarding MNH measurement [14, 32]. Furthermore, our study focused on public health settings, but many births around the world happen at home or in private facilities, including in Bangladesh where 45% of births are in private facilities [64]. Non-public service providers need to be included in both the research and policy making, especially for issues such as caesarean section, which is much higher in the private sector (Table 2) [39, 40].

Finally, we did not include service-users in our study; however, their experience of care and perceptions of data are important and under-represented in the literature. We need to better understand the potential impact of RHIS data processes on the woman and newborn’s experiences and connections with care providers; ultimately, the data belongs to them, and we must ensure that RHIS processes meet their needs and expectations (Table 2).

## Conclusion

Caesarean birth rates are rapidly increasing in all regions, with implications for data collection and use. In our study, having a caesarean changed the care pathway and affected where, what, and when data were recorded, and by whom. Mode of birth determined the location of birth (L&D ward/OT) and availability of health professionals, which impacted the data flow. Data flow and register use for caesarean births were inconsistent, leading to potential RHIS data gaps. Digitisation worsened the duplication for fragmented RHIS and increased the data burden, especially for over-stretched L&D ward staff. Health professionals were overwhelmed by duplicative RHIS tasks which competed with providing care, especially in L&D wards where there were lower staff ratios. Organisational factors were determined by facility or higher-level policies, resourcing, and culture (rather than mode of birth).

Moving forward, we need to address these challenges. Increasing the availability and quality of data (aggregate and individual-level) is necessary to inform improvements for quality of care and birth outcomes, especially given inequitable and rapid increases in caesarean births globally. The co-design of RHIS tools could help rationalise the data burden, remove duplication, increase local data ownership, and promote positive experiences for health professionals and birthing people alike.

## Supporting information

S1 Checklist Standards for Reporting Qualitative Research (SRQR) checklist. (DOCX)

S1 Text Interview guides. (DOCX)

S2 Text Reflexivity. (DOCX)

S3 Text Participant information and consent forms. (DOCX)

S4 Text Inclusivity in global research questionnaire. (DOCX)

S1 Table Ethical approval. (DOCX)

S2 Table Summary of data elements in nationally-standardised L&D registers for Bangladesh and Tanzania. (DOCX)
