# Supplementary material for: How and why does mode of birth affect processes for routine data collection and use? A qualitative study in Bangladesh and Tanzania
Source: PLOS Glob Public Health. 2024 Dec 31;4(12):e0003808. doi: 10.1371/journal.pgph.0003808 (PMC11687795; doi:10.1371/journal.pgph.0003808)
Supplement: S1 Checklist — (DOCX) [file pgph.0003808.s001.docx]

**How and why does mode of birth affect processes for routine data collection and use? A qualitative study in Bangladesh and Tanzania**

Supporting Information

# S1 Text. Interview guides

## Tanzania: All health system levels

Interviewer ID ___ ___ ___ ___

Interview date (DD/MM/YYYY): __ __ / __ __ / __ __ __ __
Location of the interview__________________________________
Name of facility: ________________________________________
Introduction: Ensure that the participant is comfortable, has time to participate in the interview.
Explain you are starting the audio recorder.
Time interview started: __________________________________
Time interview ended: __________________________________

**Section 1: Background information of the respondent:**

| Designation |  |
| --- | --- |
| Years worked in this position |  |
| Years/months worked in place/district/region/unit |  |
| Highest Education (in grade and degree) |  |
| Age |  |
| Gender |  |

**Section 2: Questions**

**Perceived utility of data**

1. Tell me what typically happens with the maternal, newborn and stillbirth data collected from health facilities? Where do these data get used?
   Probe: Facility, district, region and national level?
2. Do you think collecting maternal, newborn and stillbirth data is important? Why? Probe: Do you think some information/data are more important than others? Why? Probe: at facility, district, region, and national level?
3. Can you give an example of any information you would like to have in RHIS? What data could support improvements in the facilities?
   Probe: How do you identify data that you need? What process do you go through to get it?
4. Where do you send maternal, newborn and stillbirth data collected at the facility/district? What do they do with those data? Probe: What channel do you use to send data? How often?
5. What challenges do you face when undertaking maternal, newborn and stillbirth data related activities?

Probe: availability of guidelines, workload, indicator definitions, availability of denominator etc. How do you handle these challenges? -is there anything you think could be done to help?

1. What challenges do you face when undertaking newborn and stillbirth data related activities?
   Probe: lack of guidelines, workload, indicator definitions, availability of denominator etc. How do you handle these challenges? -is there anything you think could be done to help?
2. What is your experience of the transition between paper-based and electronic data collection?
   1. *Probe*: Does it make data collection easier or harder, why?
   2. *Probe*: Do you have the infrastructure and equipment required?

**Data demand and use**

1. How are decisions related to maternal and newborn health services made in this facility/district/region?
2. How often do you think decisions in your district/region are informed by data?
   1. *Probe:* When data is available, who in the facility/district/region reviews it?
   2. *Probe:* Who among your colleagues discuss new data or reports?
3. Has your district/region ever taken steps to improve the use of data? If so, please tell me about those efforts. Did they result in improvements for the district/region? What were the obstacles?
4. In your opinion what is the biggest obstacle to data use in your work?

**Decision making**

1. What are the different types of program decisions that are made in your facility/district/region?

*Probe:* For example, there may be decisions related to where to provide services, how to allocate resources or plan for new activities. How are decisions like these made in your facility/district/region?

*Probe:* Who is involved in the decision-making process?

*Probe:* What sources of information do you think they rely on to make decisions?

1. Was any data used for decision making? What data? Who collected it? Who analysed this data? Was the data validated or checked in any way? Do you feel comfortable and confident using data in your work?
2. Could you give me some examples of times during your work when you consulted data to inform a decision about a health service or program?
3. What specific targets are you currently tracking for your maternal and newborn–related programs?

*Probe:* How do you know when a program is not meeting these targets?

1. Does the district/region/national have plans, policies, or guidelines that relate to the collection, review or use of data?

*Probe:* Does anyone’s job description specifically address the review or use of data? What are their job titles?

1. What sources of information are needed to make decisions?
   Probe: National/regional survey data, logistics, CVRS, routine health information systems (RHIS) data etc.?

**Availability and quality**

1. Tell me about the availability of data at district/region/national level. When you need to access it for decision making, what are the process to access data, are there any challenges?
2. Can you give me an example of a time when you provided input on the design of data collection instruments?

*Probe:* Can you give me an example of a time when you provided input on the design of an indicator? i.e. MNCAH indicators?

1. Tell me about the data quality in terms of accuracy, timeliness and completeness of the information available to you from routine and non-routine sources.
2. How accurate do you think the data in your ward/facilities are? Are there any issues with maternal, newborn and stillbirth data?
3. How accurate is the information you use to monitor maternal and newborn interventions in this district/region?
4. Has the accuracy of newborn data changed over time? What influences accuracy?
5. Can you tell me about how the WHO data quality assurance tool is used for maternal and newborn data? Probe: Do you find any challenges using it? What challenge? Is it useful?
6. Who is responsible for managing data and assuring data quality within the district/region?

*Probe:* In your opinion, what are the primary causes of data quality issues?

*Probe:* How often do you perform data quality checks?

Probe: When routine supportive supervision visits are conducted, do the supervisors spend time facilitating the use of the data?

**Capacity in data use**

1. What do you think about the technical capacity within your district/region to collect, analyse, review and use data?
2. What kinds of technical assistance in routine supervision have you received in the past 6 months?

*Probe:* Who provided the technical assistance? What did they do?

**Communicating data**

1. What typically happens after the documentation of maternal, newborn and stillbirth data into the register? Do you send these data to another person/level? In what format? How often?
2. Does your district/region have a protocol, policy or written guidance for sharing or communicating data internally or externally? Would you please describe them?

*Probe*: Does sharing data include both directions from communities up to headquarters AND feedback from headquarters down to service providers?

1. Do you segment your communication to different audiences?

*Probe:* Who are your audiences for data generated by your programs?

*Probe:* How do you communicate data to your different audiences?

*Probe:* What types of information products are available to you?

*Probe:* What kinds of performance feedback does your team receive? How frequently do you receive feedback?

1. Has your district/region ever documented success stories that involved the use of data? If yes, how were these stories identified and disseminated?
2. Have they resulted in additional funding for programs, more data use activities, improvement on data quality /supervision/ etc?

**Routine data collection by mode of birth**

1. *‘’One of the findings from EN-BIRTH study phase 1, showed that data quality was lower for caesarean than vaginal births.’’*

- What do they think could cause these differences?
- Can you describe any similarities or differences between how data are collected and aggregated for caesarean compared with vaginal birth?

1. *’’We understand that women having caesarean section are cared for in a different location than women having a vaginal birth.’’*

- What are your perceptions of the data collection process for caesarean compared with vaginal births, is it the same or different?

-If so, how do you think these will impact the data quality?

- Are there any differences between the paper-based and electronic systems that are used for caesarean and vaginal births?
- What are your reflections on staff availability, knowledge, motivation (or other staff-related issues) and if these are the same or different for vaginal and caesarean births?
- How do you think staff are supported to collect data for those with vaginal compared with caesarean births, and is this the same or different
  *(By supported, we mean things like supervision, training, or feedback around MNH data quality and use)?*
- What do they think are the implications for data collection and use?
- Is there anything else they think is important to talk about?

**Effects of COVID-19 in data collection**

1. During the pandemic (especially in 2020); How Covid-19 affected the documentation process? Please explain in detail.
   Prompt: can you tell us about data flow and reporting?
2. Have you received any training on how to document any mother or child infected with Covid-19?
3. Are there any guidelines regarding routine documentation during COVID-19? If yes, please tell me in details. Are there any guidelines in this regard? If so, give details.
4. Challenges in this regard, measures taken to overcome.
5. Did you do anything to increase the number of mothers and newborn
6. Did you take any measures to achieve the target when the number of mothers and child health care recipients decreased due to Covid-19, especially during lockdown? If so, tell me more about it.
7. How would you document a mother or a child with covid-19? Covid suspected mothers – where there any formal process? How were data aggregated?

## Bangladesh: Policy-makers

Interviewer ID ___ ___ ___ ___

Interview date (DD/MM/YYYY): __ __ / __ __ / __ __ __ __
Location of the interview__________________________________
Country: _______________________________________________
Name of facility: ________________________________________
Introduction: Ensure that the participant is comfortable, has time to participate in the interview.
Explain you are starting the audio recorder.
Time interview started: __________________________________
Time interview ended: __________________________________

**Background information of the respondent:**

| Name |  |
| --- | --- |
| Designation |  |
| Years worked as health workers |  |
| Years/months worked in this facility |  |
| Years/months worked in this ward |  |
| Highest Education (in grade and degree) |  |
| Age |  |
| Gender |  |

1. **Job responsibility & Training**
   1. As a… what kind of data related activity you undertake?
   2. What challenges do you face when undertaking maternal, newborn and stillbirth data related activities?
      Probe: lack of guideline, workload etc.

Do you think these challenges differ for babies born via vaginal birth compared with caesarean birth?
How do you handle these challenges? -is there anything you think could be done to help?

- 1. Have you received any training on data collection, data analysis, data display, data reporting, using data for decision making during the past three years?

Probe: If yes, how do you feel about this training and professional development opportunities around maternal and newborn data collection and use?

Probe: Please can you give an example of what worked well or less well.
Probes: helpful, not helpful, were then any gaps?

Were there any topics you think were not necessary- if so why? How could trainings be improved?

- 1. Have you ever conducted any training on data collection, data analysis, data display, data reporting, using data for decision making during the past three years? If so, please describe your experience of providing this training.

1. **Data demand and use**
   1. Do you think collecting maternal, newborn and stillbirth data are important? Why?

Probe: Do you think some information/data are more important than others? Why?

- 1. Can you tell me what typically happens with data collected by maternal and newborn health service programs??

Probe: Who uses this information? Why? Do you think some information is more important than other? Why? Is this documentation in any way related to your own safety (from the patient party)?

- 1. How often do you think decisions are informed by DHIS2/RHIS data? Please could you share some examples?

Probe: When data are available, who in the ward/facility/district/region/unit/department reviews it?

Probe: Who among your colleagues discusses new data or reports?

- 1. What sources of information are needed to make decisions?
     Probe: National/regional survey data, logistics, CVRS, routine health information systems (RHIS) data etc.?
  2. Have you ever faced a situation that you needed to report an indicator which was not available in DHIS2? Can you give me an example of any information you would like to have? What data could support improvements in the facilities?
     Probe: How do you identify the data that you need? What process do you go through to get it?
     Probe: In your opinion what is the biggest obstacle to data use in your facility?
  3. Do you ever take any steps to improve the use of data in the health facilities? If so, please tell me about those efforts.

Probe: What were the obstacles you face? How did you mitigate for these? What do you think would help avoid this issue in the future?

In your opinion what is the opportunities and obstacles for use of DHIS2/RHIS data? How did you mitigate for these? What do you think would help avoid this issue in the future?

1. **Availability and quality**
   1. Can you access all the newborn and maternal information you need for decision making?
      Can you give us an example of when you have used data, or local information in your work with mothers and newborns? Can you access all the newborn and maternal information you need for your work?
      Probe: How easy is it to get the data from different areas and different data collection points? Have you ever faced a situation where you needed to report data on an indicator which was not available in RHIS/DHIS-2? Can you give me an example?
   2. What are the challenges do you face to collect data from DHIS2?

Probe: Unable to access server, buffering, lack of access to information

- 1. How are data collection instruments designed? Have you ever been involved in the design of data collection tools / registers?
     Probe: Can you give me an example of a time when you provided input on the design of an indicator? i.e. MNCAH indicators? Or routine data collection tool (register/tally sheet etc.)
  2. What level of input do you think it is to take policy makers input on the design of data collection instruments?
  3. Tell me about the data quality in terms of accuracy, timeliness and completeness of the information available to you from both routine and non-routine sources.
     How do you feel about the quality of DHIS2/RHIS data?
     Probe: In your opinion, what are the primary causes of data quality issues?
     Probe: Do you perform any data quality check? How? How often you do that?

What is your opinion about the RHIS data quality?
Probe: are there differences in data quality between levels of the health system?
Probe: How about between paper-based and digital records (e.g. DHIS-2)?
Probe: Or where data are collected? (e.g. L&D ward compared with the operating theatre)

- 1. Probe: In your opinion, what are the primary causes of data quality issues?
     How DHIS2/ RHIS data can be improved?
     Probe: training, logistics, management, supervision, monitoring, capacity development, reporting, resources etc
  2. What do you think the barriers and constraints to this improvement could be?
  3. Do you perform any data quality checks in your role? Are you aware of any other routine data quality checks?
     Probe: How? How often do you do that? Who?
  4. Do you ever take any steps to improve the use of data in the health facilities/policy making/decision making? If so, please tell me about those efforts.

Probe: Did you experience any obstacles? How did you mitigate for these? What do you think would help avoid this issue in the future? Did they result in improvements for the ward/facility/district/region/unit/department?

1. **Capacity in data use**
   1. What do you think about the technical capacity of the HCP/health managers to collect, analyse, review and use data?

Probe: What kind of technical capacity do you think is needed for those who use maternal, newborn and stillbirth data?

- 1. What kinds of technical assistance in routine supervision have you provided to the HCP/health manager? What did they do? Can you give an example? Is this helpful? Can you give examples of anything else you think is needed to improve capacity for newborn data collection, monitoring, and use?

1. **Communicating data**
   1. Is there any national protocol, policy or written guidance for sharing or communicating data internally or externally? Please describe them.
      Probe: Does sharing data especially maternal and newborn data include both directions, that is from communities up to headquarters AND feedback from headquarters down to service providers? Please could you share some examples of you implement this (meetings, supervision, consultations, newsletters etc.)?
   2. Do you segment your communication for maternal and newborn data to different audiences?
      Probe: Who are your audiences?
      Probe: How do you communicate data to your different audiences?
      Probe: What types of information products are available to you?
      Probe: What kinds of performance feedback does your team provide? How frequently do you provide feedback?
   3. Has your team ever documented success stories that involved the use of data? If yes, how were these stories identified and disseminated? Can you provide any examples?
   4. Have they resulted in additional funding for programs, more data use activities, improvement on data quality /supervision/ etc?
2. **Decision making**
   1. What are the different types of program decisions that are made in your district/region?

Probe: For example, there may be decisions related to where to provide services, how to allocate resources or plan for new activities. How are decisions like these made in your district/region?

Probe: Who is involved in the decision-making process?

Probe: What sources of information do you think they rely on to make decisions? Are there any important sources of data and information you think are underused? Please could you share some examples?

- 1. Could you give me some examples of times during your work when you consulted data to inform a decision about a maternal and newborn health service or program? What specific targets are you currently tracking for your maternal and newborn – related programs? And for stillbirths? How about for mode of birth (the caesarean section rate)?
     Probe: How do you set targets? How do you know when a program is not meeting these targets?
     Probe: What strategies do you use when a program is not meeting expectations/targets?
  2. Could you tell me about any current national plans, policies, procedures or guidelines that relate to the collection, review, sharing or use of data?
     Probe: internally or externally? Please describe these. Does anyone’s job description specifically address the review or use of data from DHIS2/RHIS? What are their job titles? Process of his/her work? Who is this person accountable to?

1. **Routine data collection by mode of birth**
   1. One of the findings from EN-BIRTH study phase 1, showed that data quality was lower for caesarean births, than vaginal births.
      We’d like to explore this further, what are your thoughts?

Probe: Can you describe any similarities or differences between how data are collected and aggregated for caesarean compared with vaginal birth?
Prompt: What do you think the reasons for these are?

- 1. We understand that women having caesarean section are cared for in a different location than women having vaginal birth. With this in mind what
- Do you think there any variations between the data collection processes?
  (Registers, forms, technology, location of data recording, resource availability etc.)
- Can you guide us on how you think this impacts on the data quality?
- Consider staff availability, knowledge, motivation (or other staff related issues) for those looking after women with caesarean section. Is it the same or different for those having a vaginal birth? Can you explain?
- Are they the same staff, or different?
- What do you think are the implications for data collection and use?
- Do you think there are differences in how staff are supported to collect data for those with caesarean compared with vaginal births?
  (By supported, we mean supervision, training, feedback etc. for MNH data quality, and use).
- What do you think are the implications for data collection and use?

Is there anything else you think is important that we have not talked about?

**8. Effects of COVID-19 in data collection**

8.1 How Covid-19 affected the documentation process? Please explain in detail. Have you received any training on how to document any mother or child infected with Covid-19? Are there any guidelines regarding routine documentation during COVID-19? If yes, please tell me in details. Are there any guidelines in this regard? If so, give details. Have you arranged any training for health workers in this regard? If so, please tell me in details. Challenges in this regard, measures taken to overcome

8.2. Did you take any measures to achieve the target when the number of mothers and child health care recipients decreased due to Covid-19, especially during lockdown? If so, tell me more about it.

## Bangladesh: Managers

Interviewer ID ___ ___ ___ ___

Interview date (DD/MM/YYYY): __ __ / __ __ / __ __ __ __
Location of the interview__________________________________
Country: _______________________________________________
Name of facility: ________________________________________
Introduction: Ensure that the participant is comfortable, has time to participate in the interview.
Explain you are starting the audio recorder.
Time interview started: __________________________________
Time interview ended: __________________________________

**Background information of the respondent:**

| Name |  |
| --- | --- |
| Designation |  |
| Years worked as health workers |  |
| Years/months worked in this facility |  |
| Years/months worked in this ward |  |
| Highest Education (in grade and degree) |  |
| Age |  |
| Gender |  |

1. **Job responsibility & Training**
   1. As a… what kind of data related activity you undertake?? What challenges do you face when undertaking newborn and stillbirth data related activities?
      Probe: lack of guideline, workload etc.

Do you think these challenges differ for babies born via vaginal birth compared with caesarean birth?

How do you handle these challenges? -is there anything you think could be done to help?

- 1. Have you received any training on data analysis, data display, data reporting, using data for decision making during the past three years? What was the year of the latest training?
     Probes: From where? When? Duration of training?

How do you feel about this training and professional development opportunities around maternal and newborn data collection and use? Please can you give an example Give an example of what worked well or less well.
Probes: helpful, not helpful, were then any gaps? Were there any topics you think were not necessary- if so why?

How could trainings can be improved?

- 1. Have you ever conducted any training on data collection, data analysis, data display, data reporting, using data for decision making during the past three years? If so, please describe your experience of providing this training.

1. **Data demand and use**
   1. Do you think collecting maternal, newborn and stillbirth data is important? *Why?*

Probe: Do you think some information/data are more important than others? Why?

- 1. What is your opinion regarding utility of compiled data on mothers and babies present at the wider HMIS at district/national level? Is the information enough?
     Probes: What is the role of the HMIS platform? How important is it?
  2. Can you please tell me what happens with the maternal and newborn data at the facility level after the service providers prepare and submit their monthly reports of your facility in DHIS2? Do you receive a copy of monthly reports of your facility? What measures are taken based on those reports?
     Probe: Who uses these newborn data? What information is used? What is it used for (please could you give some examples?)? (prepare data visuals or report for the comparisons among facilities in the district, comparisons with district/national targets, comparisons of data over time, comparisons of service coverage, problem identification and/or target setting, assess the health sector’s progress). Are there some data that are used more than others?
  3. Do you produce any reports or bulletins (annual, quarterly, etc.) for submission to the regional, national, global levels, based on an analysis of your routine newborn data?
     Probe: Purpose and content of these report or bulletin?
     Probe: Could do describe any additional information that would be helpful in reporting on newborn health, and stillbirths?
  4. Did you see any health workers use these data?
     Probe: Please could you give examples of why and how they used it?
     Probe: Can you describe how health workers access and use aggregated data? Do they have access in DHIS 2?
  5. Do you think decisions in your facility/district are informed by data?
     Probe: How often? When data is available, who in the facility/district reviews it?
     Probe: Who among your colleagues discuss new data or reports? Please can you give an example of any decisions informed by newborn or stillbirth related data?
  6. Has your facility/district ever taken steps to improve the use of data? If so, please tell me about those efforts.
  7. Did they result in improvements for the facility/district? What were the obstacles?
     Probe: How did you mitigate for these? What do you think would help avoid this issue in the future?
  8. Do you need any data that you don’t have?
     Probe: How do you identify data that you need? What process do you go through to get it?
     Probe: can you give me an example of any information you would like to have, that could help improve outcome for MNH in your facility?
  9. In your opinion what is the biggest obstacle to data use in your ward/facility/district/region/unit/department?
     Probe: How do you mitigate for these? What do you think would help avoid this issue in the future?
  10. What are the resource requirements for integration of the newly validated indicators in the national HMIS/DHIS2? Does the central/region/district office have the required equipment and plans for training in the use of ICT for RHIS?
      Probe: workforce, time, logistics, etc.?

1. **Availability and quality**
   1. Tell me about the availability of data within your facility/district. When you need to access it for decision making, how easy is it to do so? Can you give us an example of when you have used data, or local information in your work with mothers and newborns?

Probe: Can you access all the newborn and maternal information you need for your work? How easy is it to get the data from each facility / district. e.g. RCH department (different areas and different data collection points)? From where you get these data?

- 1. Can you give me an example of a time when you provided input on the design of data collection instruments?

Probe: Can you give me an example of a time when you provided input on the design of an indicator? i.e. MNCAH indicators?

- 1. Tell me about the data quality in terms of accuracy, timeliness and completeness of the information available to you from both routine and non-routine sources.

Who is responsible for managing data and assuring data quality within the facility/district?
Probe: In your opinion, what are the primary causes of data quality issues?
Probe: How often do you perform data quality checks? How it was done? Can you give an example? How often was it checked? Do you maintain a record of data quality assessment?
Probe: When routine supportive supervision visits are conducted do the supervisors spend time on facilitating the use of the data?

- 1. What is your opinion about the RHIS data quality?
     Probe: are there differences in data quality between levels of the health system?
     Probe: How about between paper-based and digital records (e.g. DHIS-2)?
     Probe: Or where data are collected? (e.g. L&D ward compared with the operating theatre)

Probe: In your opinion, what are the primary causes of data quality issues?

- 1. Do you perform any data quality checks in your role? Are you aware of any other routine data quality checks?
     Probe: How? How often do you do that? Who?
  2. Do you ever take any steps to improve the use of data in the health facilities/policy making/decision making? If so, please tell me about those efforts.

Probe: Did you experience any obstacles? How did you mitigate for these? What do you think would help avoid this issue in the future? *Did they result in improvements for the ward/facility/district/region/unit/department?*

- 1. Can you describe any situation, where the health worker noticed any issues with their own documentation quality? Please explain.
     Probe: What information? What issues?
  2. What are the opportunities to improve the data collection and use at different layers? How can we improve the routine information system? What kind of resources you feel necessary for this improvement?
     Probes: What could be the barriers and constraints to this improvement?
  3. Do you ever get any feedback from the data that you send up the health system
     Probes: How often? Who provides the feedback?
  4. Are there any written guidelines on RHIS information display, use, and feedback?
     Probe: discussion with staff, were any decisions made based on the discussion, follow up of the decision.

1. **Capacity in data use**
   1. Do you have any training on usage or feedback of these data? From where? When? Duration of training?
      Probe: How do you feel about this training? Give an example.
      Probes: helpful, not helpful
   2. What do you think about the technical capacity the HCP within your facility/district to collect, analyse, review and use data?
   3. What kinds of technical assistance in routine supervision have you provided in to the HCP?
      Probe: Who provided the technical assistance? What did they do? Can you give an example?
      Is this helpful? Can you give examples of anything else you think is needed to improve capacity for newborn data collection, monitoring and use?
2. **Communicating data**
   1. Does your facility/district have a protocol, policy or written guidance for sharing or communicating data internally or externally? Please describe them.
      Probe: Does sharing data especially maternal and newborn data include both directions, that is from communities up to headquarters AND feedback from headquarters down to service providers?
      Probe: Please could you share some examples of you implement this (meetings, supervision, consultations, newsletters etc.)?
   2. Do you segment your communication for maternal and newborn data to different audiences?
      Probe: Who are your audiences for data generated by your programs?
      Probe: How do you communicate data to your different audiences?
      Probe: What types of information products are available to you?
      Probe: What kinds of performance feedback does your team receive? How frequently do you receive feedback?
   3. Have you facility/district ever documented success stories that involved the use of data? If yes, how were these stories identified and disseminated? Can you provide any examples?
   4. Have they resulted in additional funding for programs, more data use activities, improvement on data quality /supervision etc.?
3. **Decision making**
   1. What are the different types of program decisions (maternal and child health, and specifically for newborn and stillbirths) that are made in your facility /district?
      Probe: For example, there may be decisions related to where to provide services, which area should focus, how to allocate resources or plan for new activities. How are decisions like these made in your facility?
      Probe: Who is involved in the decision-making process?
      Probe: What sources of information do you rely on to make decisions?
      Probe: Are there any important sources of data and information you think are underused? Please could you share some examples?
   2. Could you give me some examples of times during your work when you consulted data to inform a decision about a newborn health service or program?
   3. What specific targets are you currently tracking for your maternal and newborn and specifically for newborn and stillbirth – related programs? How do you get these targets?
      Probe: How do you know when a program is not meeting these targets? How about for mode of birth (the caesarean section rate)?
      Probe: If you are aware that a program in not meeting expectations, what kinds of things can you do about it?
   4. Could you tell me about any current national plans, policies, procedures or guidelines that relate to the collection, review or use of data you use in your facility?
      Probe: internally or externally? Please describe these. Does anyone in your facility have job description specifically addresses the review or use of data? What are their job titles? Process of his/her work? Who is this person accountable to? How long does it take? How often?
   5. What do you think the duration of data extraction should be?
4. **Routine data collection by mode of birth**
   1. One of the findings from EN-BIRTH study phase 1, showed that data quality was lower for caesarean births, than vaginal births.
      We’d like to explore this further, what are your thoughts?

Prompt: Can you describe any similarities or differences between how data are collected and aggregated for caesarean compared with vaginal birth?
Prompt: What do you think the reasons for these are?

- 1. We understand that women having caesarean section are cared for in a different location than women having vaginal birth. With this in mind what
- Do you think there any variations between the data collection processes?
  *(Registers, forms, technology, location of data recording, resource availability etc.)*
- Can you guide us on how you think this impacts on the data quality?
- Consider staff availability, knowledge, motivation (or other staff related issues) for those looking after women with caesarean section. Is it the same or different for those having a vaginal birth? Can you explain?
- Are they the same staff, or different?
- What do you think are the implications for data collection and use?
- Do you think there are differences in how staff are supported to collect data for those with caesarean compared with vaginal births?
  *(By supported, we mean supervision, training, feedback etc. for MNH data quality, and use).*
- What do you think are the implications for data collection and use?

Is there anything else you think is important that we have not talked about?

1. **Effects of COVID-19 in data collection**
   1. How Covid-19 affected the documentation process? Please explain in detail. Have you received any training on how to document any mother or child infected with Covid-19? Are there any guidelines regarding routine documentation during COVID-19? If yes, please tell me in details. Are there any guidelines in this regard? If so, give details. Have you arranged any training for health workers in this regard? If so, please tell me in details. Challenges in this regard, measures taken to overcome
   2. Did you take any measures to achieve the target when the number of mothers and child health care recipients decreased due to Covid-19, especially during lockdown? If so, tell me more about it.

## Bangladesh: Healthcare workers

**Advancing Routine Health Management Information Systems (HMIS) to deliver for Every Newborn (EN-BIRTH study phase 2): Assessing the feasibility of incorporating, and exploring data quality and utility for selected maternal and newborn indicators in Kushtia, Bangladesh**

**Key informant interview guide: for Health Care Provider**

Interviewer ID ___ ___ ___ ___

Interview date (DD/MM/YYYY): __ __ / __ __ / __ __ __ __
Location of the interview__________________________________
Country: _______________________________________________
Name of facility: ________________________________________
Introduction: Ensure that the participant is comfortable, has time to participate in the interview.
Explain you are starting the audio recorder.
Time interview started: __________________________________
Time interview ended: __________________________________

**Background information of the respondent:**

| Name: |  |
| --- | --- |
| Designation: |  |
| Years worked as health workers |  |
| Years/months worked in this facility |  |
| Years/months worked in this ward |  |
| Highest Education (in grade and degree) |  |
| Age |  |
| Gender |  |

1. **Job responsibility & Training**
   1. What are your job responsibilities? How do you feel about your job responsibility?
      Probe: Health service activities, Support activities, Additional activities
   2. Have you received any training on data collection, data analysis, data display, data reporting, using data for decision making during the past three years? What was the year of the latest training?
      Probes: From where? When? Duration of training?
   3. How do you feel about this training? Give an example of what worked well or less well.
      Probes: helpful, not helpful, were then any gaps? Were there any topics you think were not necessary- if so why?
   4. How could trainings be improved?
2. **Data demand and use**
   1. Do you think collecting maternal and child health data is important? Why?

Probe: Do you think some information/data are more important than others? Why?

- 1. Can you tell me what typically happens with the data collected by you?
     Probes: Who uses the data? What are they used for? What information is used? Are some data more valuable than others? Why?
  2. How often do you think decisions in your facility are informed by the data you collected?
  3. Does your facility need data that you don’t have?
     Probe: can you give me an example of any information you would like to have? What data could support improvements in your facility?
     Probe: How do you identify data that you need? What process do you go through to get it?
     Probe: In your opinion what is the biggest obstacle to data use in your facility?
  4. Has your facility ever taken steps to improve the use of data? If so, please tell me about those efforts.
     Did they result in improvements for the facility? What were the obstacles?
     Probe: what do you think could help increase the use of data in your facility?

1. **Availability and quality**
   1. Can you access all the maternal and child health information you need for your work? Do you use these data? How do you use these data? When you need to access it, how easy is it to do so?
      Probe: From where you get these data? access in DHIS 2 or hard copy
   2. Have you ever been involved in the design of data collection tools / registers? Please share an example.
   3. How important do you think it is to take HCP’s input on the design of data collection instruments?
   4. Would you please share your understanding and experiences regarding data quality? Tell me about the data quality in terms of accuracy, timeliness and completeness of the information available to you from both routine and non-routine sources.
      What do you do for managing data and assuring data quality within the facility?
      Probe: In your opinion, what are the primary causes of data quality issues?
   5. Do you ever take any steps to improve the use of data in the health facilities/policy making/decision making? If so, please tell me about those efforts.

Probe: Did you experience any obstacles? How did you mitigate for these? What do you think would help avoid this issue in the future? Did they result in improvements for the ward/facility/district/region/unit/department? How do you think data quality can be improved?

- 1. What could be the barriers and constraints to this improvement?
  2. How do you ensure your own data quality? How often does anyone perform data quality checks on your data? How did you feel about the whole quality checking procedure?
     Probe: When routine supervision visits are conducted, do the supervisors spend time on facilitating the use of the data?
     Probe: Could you share an example?

Probe: What do supervisors generally focus on?

- 1. What challenges do you face when undertaking maternal and child health data related activities?
     Probe: lack of guideline, workload etc. How do you handle these challenges? Is there anything you think could be done to help?

Probe: Do you think these challenges differ for babies born via vaginal birth compared with caesarean birth?

1. **Capacity in data use**
   1. What kinds of technical assistance in routine supervision have you received in the past 6 months?
      Probe: Who provided the technical assistance? What did they do?
      Probe: Did you find it helpful?

Probe: Can you give an example?

- 1. What do you think about the technical capacity within your ward/facility/district/region/unit/department to collect, analyse, review and use data?

1. **Communicating data**
   1. Does your facility have a protocol, policy or written guidance for sharing or communicating data internally or externally? Please describe them.
      Probe: Newborn data guideline, maternal data guideline, etc. Does sharing data include both directions; that is from communities up to headquarters and feedback from headquarters down to service providers?
   2. Do you segment your communication to different audiences?
      Probe: Who are your audiences for data generated by your programs?
      Probe: How do you communicate data to your different audiences?
      Probe: What types of information products are available to you?
      Probe: What kinds of performance feedback does you receive? From whom? How frequently do you receive feedback?
   3. Has your facility ever documented success stories that involved the use of data? If yes, how were these stories identified and disseminated?
      Probe: Can you provide any examples?
   4. Have they resulted in additional funding for programs, more data use activities, improvement on data quality /supervision etc.?
2. **Decision making**
   1. How do you think maternal and child health decisions are made in your facility?

Probe: For example, there may be decisions related to where to provide services, how to allocate resources or plan for new activities.
Probe: Who is involved in the decision-making process? How do they take these decisions?
Probe: What sources of information do you think they rely on to make decisions? Whether the collected data are used?

- 1. Could you give me some examples of times during your work when you consulted data to inform a decision about a newborn health service or program?

1. **Routine data collection by mode of birth**
   1. Do you currently care for women and newborns having both vaginal and caesarean births? If so, please could you describe your usual scope of work?
   2. One of the findings from EN-BIRTH phase 1 suggests there is a difference in data collection and flow between vaginal and caesarean births. We’d like to explore this further, what are your thoughts?

We’d like to explore this further, what are your thoughts?

Prompt: Can you describe any similarities or differences between how data are collected and aggregated for caesarean compared with vaginal birth?
Prompt: What do you think the reasons for these are?

- 1. We understand that women having caesarean section are cared for in a different location than women having vaginal birth. With this in mind what
- Do you think there any variations between the data collection processes?
  *(Registers, forms, technology, location of data recording, resource availability etc.)*
- Can you guide us on how you think this impacts on the data quality?
- Consider staff availability, knowledge, motivation (or other staff related issues) for those looking after women with caesarean section. Is it the same or different for those having a vaginal birth? Can you explain?
- Are they the same staff, or different?
- What do you think are the implications for data collection and use?
- Do you think there are differences in how staff are supported to collect data for those with caesarean compared with vaginal births?
  *(By supported, we mean supervision, training, feedback etc. for MNH data quality, and use).*
- What do you think are the implications for data collection and use? Is there anything else you think is important that we have not talked about?

1. **Effects of COVID-19 in data collection**
   1. How Covid-19 affected the documentation process? Please explain in detail. Have you received any training on how to document any mother or child infected with Covid-19? Are there any guidelines regarding routine documentation during COVID-19? If yes, please tell me in details. Are there any guidelines in this regard? If so, give details. Have you arranged any training for health workers in this regard? If so, please tell me in details. Challenges in this regard, measures taken to overcome

Did you take any measures to achieve the target when the number of mothers and child health care recipients decreased due to Covid-19, especially during lockdown? If so, tell me more about it.
