# Supplementary material for: How and why does mode of birth affect processes for routine data collection and use? A qualitative study in Bangladesh and Tanzania
Source: PLOS Glob Public Health. 2024 Dec 31;4(12):e0003808. doi: 10.1371/journal.pgph.0003808 (PMC11687795; doi:10.1371/journal.pgph.0003808)
Supplement: S1 Text — (DOCX) [file pgph.0003808.s002.docx]

**How and why does mode of birth affect processes for routine data collection and use? A qualitative study in Bangladesh and Tanzania**

Supporting Information

# S2 Text. Reflexivity

Reflexivity describes the ongoing reflective process by which researchers evolve self-awareness and interrogate their own role in data collection and interpretation (Symon & Cassell, 2012). Researcher positioning is dynamic, and I recognise that my own, and my colleagues, positioning interacts with the knowledge production during this study (Bishop & Shephard, 2011). I am a British mother and a midwife with experience working clinically in a range of high and low-income settings. I enjoy deep topic insight through my experience collecting and using routine data as both a practitioner and a researcher. I experience the duality of being ‘subject to’ data collection and/or ‘imposing’ data collection on others. I have these insider perspectives of the phenomena we are investigating, but I’m also an outsider within our study contexts.

This work has been undertaken by a multiprofessional, multicounty team who have also supported me to lead the analysis for this paper as part of my PhD. The three team members (HR, TM, DS) most closely involved throughout all stages of this qualitative study, have met regularly to plan, collect, and analyse the data. These team meetings have been integral to fostering an equitable partnership built on a deep respect for the local knowledge and expertise held by TM & DS; all considering the legacy of colonial injustice and power imbalances. Meetings were most frequent during data collection and analysis, especially as themes/codes were being identified and data interpreted. The transcripts were coded by two researchers (internal and external) (HR, TM, DS) to check the reliability of coding and promote opportunities to interrogate interpretation.

We undertook this phenomenological qualitative research within a constructivist paradigm using in-depth interviews. This research aimed to explore the realities of how mode of birth affects data and collection and use for healthcare and data professionals working with at all levels of the public health care systems in Bangladesh and Tanzania. We therefore used a constructivist approach to facilitate exploration of the subjective interpretations and meanings that participants attach to various aspects of routine data collection and the perceived influence of mode of birth. Using this phenomenological approach, we were able to investigate how the context (e.g., the hospital environment, professional guidelines, cultural attitudes towards different birth methods) influenced participants practices and decisions around routine data collection and if/how this was affected by mode of birth (Neubauer et al., 2019). As suggested by Peck and Mummery (2023), application of this lens during our analysis enabled us to explore how participants perceived the potential impact of institutional norms, cultural/social/political attitudes, and their own inherent assumptions more deeply, contributing to the credibility of our findings.

We used cases to explore themes and exceptions in the data (situations where an account appeared to contradict the emerging theme). Alternatively, there were also several accounts where the participant’s described intention contradicts their actions. Or, from a remote community setting where the participant could easily manage all their tasks (but had a low through-put of cases). Although the analysis predominately compared individual accounts to each other (to identify themes across multiple accounts), we also interrogated the individual accounts in relation to specific incidents of disclosure. We further compared the accounts of groups of participants to explore potential similarities or differences based on a range of different characteristics (e.g. role, age, level of the health system, seniority etc). The interviews were generally conducted face to face in the participants’ workplace (as their preferred location), but this created a sense of urgency in many of the interviews. The researchers tried to be respectful of participant’s time but were often aware that the participant felt time pressured. As a team, we discussed the potential impact of the researcher’s own status and perceived priorities on the participants accounts. We tried to mitigate the risk that participants would adapt their testimony to please the researchers who they could recognise as valuing RHIS data, or as being more senior, or having connections with *higher* authorities. Confidentiality was reiterated and suggestions given for potentially more private interview locations when necessary. This necessitated consideration when interpreting the data.

The wider research team and my PhD committee have supported in crucial moments drawing on their expertise, knowledge of the setting/methods to discuss the emerging themes, potential lines of new enquiry, and interpretation. High-income country researchers are over-represented on the list of authors because the work has been undertaken as part of my PhD in a UK-based institution under the supervision of white western academics (although one born in Uganda). We acknowledge the EN-BIRTH-2 study team in this paper; it has been drafted as part of a collaborative authorship agreement that also includes publications led by Bangladeshi and Tanzanian colleagues. For this paper, the named authors are predominately female (10/13), 7 are from LMICs. There was intentional inclusion of early career researchers (just 3 named Professors: SEA, JS, JEL). I’m not sure if that’s why female authors are heavily represented in the group.

The project is fostering opportunities for two PhD candidates, myself and Donat Shamba (from IHI, Tanzania). This does not exonerate my privilege. I am also implicated in the institutions that perpetuate inequities through parachute research, including LSTHM which has a long colonial history. I am committed to challenging the structures and status quo. Personally, I have engaged reflexively throughout the research process by interrogating my assumptions about global partnerships, the participants, the personal and professional values that underpin my own experiences and practice, and how these assumptions might affect or shape my understanding of the study data (Berger, 2015). This includes a recognition of the hierarchies inherent in health system structures, funding, and data systems (as well as academia).

**Covid Pandemic Impact Statement**
The pandemic delayed data collection in Tanzania. In Bangladesh, data collection was able to go ahead (Sept-Dec 2020) in accordance with social distancing guidelines as lockdown restrictions had eased. Potential challenges might have included maternity staff being redeployed to support with pandemic response, maternity services being closed due to overburdening of the health system etc. However, this wasn’t reported in our study settings.

**References**:

Berger, R. (2015). Now I see it, now I don’t: Researcher’s position and reflexivity in qualitative research. Qualitative research, 15(2), 219-234.

Bishop, E. C., & Shepherd, M. L. (2011). Ethical reflections: Examining reflexivity through the narrative paradigm. Qualitative Health Research, 21(9), 1283-1294.

Symon, G., & Cassell, C. (2012). Qualitative organizational research: core methods and current challenges. Sage.

Neubauer BE, Witkop CT, Varpio L. How phenomenology can help us learn from the experiences of others. Perspect Med Educ. 2019 Apr;8(2):90-97. doi: 10.1007/s40037-019-0509-2. PMID: 30953335; PMCID: PMC6468135.

Peck, B., & Mummery, J. (2023). Hermeneutic Constructivism: One ontology for authentic understanding. Nursing Inquiry, 30, e12526. https://doi.org/10.1111/nin.12526
