# Supplementary material for: How and why does mode of birth affect processes for routine data collection and use? A qualitative study in Bangladesh and Tanzania
Source: PLOS Glob Public Health. 2024 Dec 31;4(12):e0003808. doi: 10.1371/journal.pgph.0003808 (PMC11687795; doi:10.1371/journal.pgph.0003808)
Supplement: S2 Text — (DOCX) [file pgph.0003808.s003.docx]

**How and why does mode of birth affect processes for routine data collection and use? A qualitative study in Bangladesh and Tanzania**

Supporting Information

# S3 Text. Participant information and consent forms

## Tanzania

The consent process undertaken in the language of the participant’s choice. Swahili translations are available upon request.

**Qualitative Key Informant Interviews**

**Protocol Title:** Advancing Routine Health Management Information Systems (HMIS) to deliver for Every Newborn - EN-BIRTH study phase 2

**Principle Investigator's names:**

Dr Honorati Masanja, Ifakara Health Institute (IHI)

Dr Shams El Arifeen, International centre for diarrheal disease research, Bangladesh (icddr,b),

Prof Joy Lawn and Dr Louise T Day, London School of Hygiene & Tropical Medicine (LSHTM)

Dr Kavita Singh, Data for Impact (D4I)

**Participant information**

**Purpose of the research:**

We work at Ifakara Health Institute (IHI) in collaboration with the London School of Hygiene & Tropical Medicine (LSHTM), International centre for diarrheal disease research, Bangladesh (icddr,b), and Data for Impact (D4I), we are conducting a study to assess the feasibility, data quality and utility of incorporating selected maternal and newborn indicators within national routine HMIS/DHIS2. Findings from this study will help us in understanding the feasibility of incorporating selected indictors, for tracking progress towards meeting the sustainable development goal (SDG) targets for maternal and newborn health.

**Why we have selected you?**

We are inviting you to participate in this study because you are involved with data collection and use relevant to maternal and newborn health services.

**Methods and Procedures:**

If you agree to take part in the study, you may be asked to participate in the following activities:

- One to one survey or interview(s)
- Focus group or discussion(s)

**Risks and benefits:**

This is an observational study and we therefore do not anticipate increased risks related to your participation in this study. Neither your name nor the names of any other participants in this study will be included in the data set or in any report. Participation in this study may not benefit you directly. The information that we will obtain will be very useful to help improve the newborn health care delivery system in Bangladesh and Tanzania.

All research involving human participants is looked at by an independent group of people, called a Research Ethics Committee, to protect your interests. This study has been reviewed by the following committees: IHI, Icddr,b, LSHTM, and D4I. These Ethics Committees have reviewed the study and have agreed that it is okay for us to ask people to take part.

**Privacy, anonymity, and confidentiality:**

IHI in collaboration with the LSHTM, icddr,b and D4I, have responsibility for the collection, storage, analysis and use of your data. We do hereby affirm that privacy, anonymity and confidentiality of information identifying you will strictly be maintained, and any information about you which leaves the facility/research study group, will have your name and address removed so that you cannot be recognised. We would keep your personal details confidential. None other than the investigators will have access to this information which will be kept in a different safe place to the other study information and will be destroyed after 10 years. We would be happy to answer any questions about the study.

**Future use of information:**

Anonymous or abstracted information and data will be archived and made available with other researchers within and outside the country. However, this will not conflict with or violate the maintenance of privacy, anonymity and confidentiality of information identifying participants in any way. The study results will be published in a medical journal so that other public health professionals can learn from them. Your personal information will not be included in the study report.

**Right not to participate and withdraw:**

Your participation in the study is voluntary, and you have the sole authority to decide for or against your participation. You would also be able to withdraw from participation any time during the study, without showing any cause. Refusal to participate in the study will not cause you any harm. We may visit you again later, for a second visit, if needed. If you agree to the first visit, you do not have to agree to the second.

**Principle of compensation:**

As mentioned earlier, your participation in this study is completely voluntary and you will not get any payment for participating in this study.

**Contact person:**

If you have questions about this study or if you feel that you have been treated unfairly or have been hurt by joining the study, you may communicate with the following personnel:

| Purpose of contact | Name and address | Address for communication |
| --- | --- | --- |
| For any question related to the study, or any problem | Dr. Honorati Masanja  IHI, Principle investigator | [hmasanja@ihi.or.tz](mailto:hmasanja@ihi.or.tz)  +255 22 2774756 |
| To know about research ethics | Dr. Mwifadhi Mrisho  IHI IRB secretary | [mmrisho@ihi.or.tz](mailto:mmrisho@ihi.or.tz)  +255 788766676 |

If you agree to our proposal of enrolling in our study, please indicate that by putting your signature at the specified space below. Thank you for your cooperation

**Participant:** I certify that

- I have had the information explained to by study personnel in a language that I understand.
- I have had the opportunity to consider the information, ask questions and have these answered satisfactorily.
- I understand that my participation is voluntary and that I am free to withdraw at any time without giving any reason, without my legal rights being affected.
- I understand that the interview will be recorded, transcribed, de-identified and stored.
- I understand that these data may be shared via a public data repository or by sharing directly with other researchers, and that I will not be identifiable from this information
- I understand that anonymized quotes from this interview may be extracted and used as part of the research outputs.

___________________ __________________

Signature/thumb print of participant Date

___________________ __________________

Signature of impartial witness (non-family member) Date

___________________ __________________

Signature of interviewer Date

## Bangladesh

The consent process undertaken in the language of the participant’s choice. Bangla translations are available upon request.

**Qualitative Key Informant Interviews**

**Protocol Title:** Advancing Routine Health Management Information Systems (HMIS) to deliver for Every Newborn - EN-BIRTH study phase 2

**Principle Investigator's names:**

Dr Honorati Masanja, Ifakara Health Institute (IHI)

Dr Shams El Arifeen, International centre for diarrheal disease research, Bangladesh (icddr,b),

Prof Joy Lawn and Dr Louise T Day, London School of Hygiene & Tropical Medicine (LSHTM)

Dr Kavita Singh, Data for Impact (D4I)

**Participant Information Sheet**

**Purpose of the research:**

We work at icddr,b which was previously known as International Centre for Diarrheal Diseases Research, Bangladesh. In collaboration with the London School of Hygiene & Tropical Medicine (LSHTM), Ifakara Health Institute (IHI), Data for Impact (D4I), and Research for Decision Making (RDM) activity of USAID, we are conducting a study to assess the feasibility, data quality and utility of incorporating selected maternal and newborn indicators within national routine HMIS/DHIS2. As a part of this research, we need to observe service delivery activities of health workers at selected public health facilities in Bangladesh. Findings from this study will help us in understanding the feasibility of incorporating selected indictors, for tracking progress towards meeting the sustainable development goal (SDG) targets for maternal and newborn health.

**Why we have selected you?**

We are inviting you to participate in this study because you are involved with data collection and use of relevant maternal and newborn health indicators.

**Methods and Procedures:**

If you agree to take part in the study, you may be asked to participate in the following activities:

- One to one survey or interview(s)
- Focus group or discussion(s)

**Risks and benefits:**

This is an observational study and we therefore do not anticipate increased risks related to your participation in this study. Neither your name nor the names of any other participants in this study will be included in the data set or in any report. Participation in this study may not benefit you directly. The information that we will obtain will be very useful to help improve the newborn health care delivery system in Bangladesh and Tanzania.

All research involving human participants is looked at by an independent group of people, called a Research Ethics Committee, to protect your interests. This study has been reviewed by the following committees: icddr,b (<20015 >), LSHTM, IHI and D4I, These Ethics Committees have reviewed the study and have agreed that it is okay for us to ask people to take part.

**Privacy, anonymity and confidentiality:**

icddr,b in collaboration with the LSHTM, IHI and D4I, have responsibility for the collection, storage, analysis and use of your data. We do hereby affirm that privacy, anonymity and confidentiality of information identifying you will strictly be maintained, and any information about you which leaves the facility/research study group, will have your name and address removed so that you cannot be recognised. We would keep your personal details confidential. None other than the investigators will have access to this information, which will be kept in a different safe place to the other, study information and will be destroyed after 10 years. We would be happy to answer any questions about the study.

**Future use of information:**

Anonymous or abstracted information and data will be archived and made available with other researchers within and outside the country. However, this will not conflict with or violate the maintenance of privacy, anonymity and confidentiality of information identifying participants in any way. The study results will be published in a medical journal so that other public health professionals can learn from them. Your personal information will not be included in the study report.

**Right not to participate and withdraw:**

Your participation in the study is voluntary, and you have the sole authority to decide for or against your participation. You would also be able to withdraw from participation any time during the study, without showing any cause. Refusal to participate in the study will not cause you any harm. We may visit you again later, for a second visit, if needed. If you agree to the first visit, you do not have to agree to the second.

**Principle of compensation:**

As mentioned earlier, your participation in this study is completely voluntary and you will not get any payment for participating in this study.

**Contact person:**

If you have questions about this study or if you feel that you have been treated unfairly or have been hurt by joining the study, you may communicate with the following personnel:

| Purpose of contact | Name and address | Address for communication |
| --- | --- | --- |
| For any question related to the study, or any problem | Dr. Shafiqul Ameen, Research Investigator, Maternal and child health division (MCHD) | Address: icddr,b, Mohakhali, Dhaka-1212 Phone no.: 01711389302 (9:00 am to 5:00 pm |
|  | Dr. Ahmed Ehsanur Rahman, Associate Scientist, Maternal and child health division (MCHD) | Address: icddr,b, Mohakhali, Dhaka-1212 Phone no.: 01727010050 (9:00 am to 5:00 pm |
| To know the rights or benefits or to log any complain or dissatisfaction | M A Salam Khan (IRB Coordinator) | IRB Secretariat, Research Administration, icddr,b, Mohakhali, Dhaka-1212 Phone: (+88-02) 982708 |

If you agree to our proposal of enrolling in our study, please indicate that by putting your signature at the specified space below. Thank you for your cooperation

**Participant:**

I certify that

- I have had the information explained to by study personnel in a language that I understand.
- I have had the opportunity to consider the information, ask questions and have these answered satisfactorily.
- I understand that my participation is voluntary and that I am free to withdraw at any time without giving any reason, without my legal rights being affected.
- I understand that the interview will be recorded, transcribed, de-identified and stored.
- I understand that these data may be shared via a public data repository or by sharing directly with other researchers, and that I will not be identifiable from this information
- I understand that anonymized quotes from this interview may be extracted and used as part of the research outputs.

___________________ __________________

Signature/thumb print of participant Date

___________________ __________________

Signature of impartial witness (non-family member) Date

___________________ __________________

Signature of interviewer Date
