# Supplementary material for: How and why does mode of birth affect processes for routine data collection and use? A qualitative study in Bangladesh and Tanzania
Source: PLOS Glob Public Health. 2024 Dec 31;4(12):e0003808. doi: 10.1371/journal.pgph.0003808 (PMC11687795; doi:10.1371/journal.pgph.0003808)
Supplement: S1 Table — (DOCX) [file pgph.0003808.s006.docx]

**How and why does mode of birth affect processes for routine data collection and use? A qualitative study in Bangladesh and Tanzania**

Supporting Information

# S1 Table. Ethical approval

| **Institution** | **Date(s) granted** | **Number/ref** |
| --- | --- | --- |
| London School of Hygiene & Tropical Medicine (LSHTM) | 04.6.21  20.06.22  06.06.23  28.05.24 | 22081 (EN-BIRTH-2)  27907 (PhD)  Continuation (12 months)  Continuation (12 months) |
| Icddr,b Bangladesh Research review Committee | 05.04.20  04.04.21 | PR 20015  Continuation (12 months) |
| Ifakara Health Institute, Tanzania | 01.03.21  27.02.23  28.05.24 | IHI/IRB/No: 10-2021  Continuation (12 months)  Continuation (12 months) |
| National Institute for Medical Research (NIMRI), Tanzania | 22.04.21  09.05.22  19.02.24 | NIMR/HQ/R.8c/Vol.IX/3662  Extension (12 months)  Extension (12 months) |
