# Supplementary material for: How and why does mode of birth affect processes for routine data collection and use? A qualitative study in Bangladesh and Tanzania
Source: PLOS Glob Public Health. 2024 Dec 31;4(12):e0003808. doi: 10.1371/journal.pgph.0003808 (PMC11687795; doi:10.1371/journal.pgph.0003808)
Supplement: S2 Table — (DOCX) [file pgph.0003808.s007.docx]

**How and why does mode of birth affect processes for routine data collection and use? A qualitative study in Bangladesh and Tanzania**

Supporting Information

# S2 Table. Summary of data elements in nationally-standardised L&D registers for Bangladesh and Tanzania

|  | **Bangladesh, National EmONC Register**  **(DGFP & DGHS)** | **Tanzania, MTUHA 12 Register** |
| --- | --- | --- |
| **Language** | **Bangla (unless stated)** | **Swahili (unless stated)** |
| **ADMIN** |  |  |
| **Identification #** | Free text | Free text |
| **Name** | Free text | Free text |
| **Contact details** | Free text in DGHS/ For DGFP, write: Father/husbands name, address, mobile number | Free text |
| **Date and Time of Admission** | Free text | Free text |
| **Age**  *(can disaggregate adolescents)* | Free text | Free text |
| **How many pregnancies (gravida)** | Free text | Free text |
| **How many times did she give birth (Parity)** | Free text | Free text |
| **Number of Children living** | **No column** | Free text |
| **LMP**  *(Last monthly period)* | DGFP: Free text (no column in DGHS) | **No column** |
| **# of ANC received: First contact (ANC1), at least 4 contacts (ANC4+), at least 8 contacts (ANC8+)** | Free text | **No column** |
| **INTERVENTIONS** |  |  |
| **ACS given (24-34/40)** | Tick if given between 24-34 weeks | **No column** |
| **Location of birth (home/ TBA/ facility)** | **No column** | Write: HF (health facility), BBA (born Before arrival to facility), TBA (traditional birth attendant), H (Nyumbani). Or home, all other abbreviations use English language |
| **Who conducted the delivery (SBA)** | Who conducted the delivery or operation  Tick: Doctor, family welfare officer, midwife, nurse, other (Bangla) | Write: Name, cadre |
| **Use of partograph** | Free text (ticked if used) | **No column** |
| **Type of anaesthesia** | For DGFP only, tick: G/A, SAB, Epidural, Ketamine, Others (list in English) (no column in DGHS) | **No column** |
| **Uterotonics for the prevention of PPH** | Free text box for AMTST: 1. Administration of uterotonic within 1 minute of delivery; 2. Controlled traction of umbilical cord for delivery of placenta and 3. Uterine massage (Ticked if done) (using English language abbreviation) | (a) Has been given Oxytocin write "O" or Ergometrine "E" or Misoprostol "M" |
|  |  | (b) Expulsion of the placenta by pulling the umbilical cord slowly: Write Yes "N" or No "H." |
|  |  | (c) Uterine massage: Write Yes "N" or No "H." |
| **Treatment of PPH** | Free text box; 'Medicine given' | Oxytocin/ergometrine/misoprostol: Yes N" or No "H." |
| **Provision of antibiotics (maternal)** | Free text box; 'Medicine given' | Write yes "N" or No "H." |
| **Provision of magnesium sulphate** | Free text box; 'Medicine given' | Write yes "N" or No "H." |
| **Manual removal of placenta** | Free text box; 'Any other operation' | Write yes "N" or No "H." |
| **MVA (Manual Vacuum Aspiration) or D&C (Dilation and Curettage)** | Free text box; 'Any other operation' | Write yes "N" or No "H." |
| **Blood transfusion** | Free text (Ticked if given) | Write yes "N" or No "H." |
| **Newborn was dried within 1 minute of birth** | Free text (Ticked if yes). | **No column** |
| **Newborn didn't cry/ breath immediately after birth without stimulation** | Tick: Yes, No. | **No column** |
| **Neonatal resuscitation** | Free text (Ticked if yes). | Write "1" suction, "2" stimulation, "3" bag and mask, and "H" no |
| **7.1% Chlorohexidine used for umbilical cord** | Free text (Ticked if yes). | **No column** |
| **Newborn was given skin to skin care by mother** | Free text (Ticked if yes). | **No column** |
| **Breastfed within 1 hour of birth** | Free text (Ticked if yes). | Write yes "N" or No "H." |
| **Exclusive Breastfeeding** | **No column** | Write EBF = Exclusive Breastfeeding, RF = Replacement Feeding (using English language abbreviation) |
| **Family Planning Counselling** | **No column** | Write 1 = New, 2 = Repeat/Returning |
| **Contraceptive method provided** | DGHS, Free text. DGFP, tick: Advice given, procedure, oral contraceptive, IUD, implant, tubectomy | Write (condom "KO", pills "POP", injection "S", Jadelle "JD", Implanon "IM", IUD "KT", tubal ligation "BTL" |
| **HIV testing during labour and after delivery** | **No column** | Write P, N, U. "P" Positive, "N" Negative, "U" Unknown." (abbreviation uses English word but explanation is in Swahili) |
| **ARV medication (if mother HIV positive).** | Free text box; 'Medicine given' | Write N = has been given, H = has not been given |
| **Postnatal check 1 Mother** | Free text (ticked if provided) | **No column** |
| **Postnatal check 2 Mother** | Free text (ticked if provided) | **No column** |
| **Postnatal check 1 Newborn** | Free text (ticked if provided) | Assessment of the child after 24 hours: Write "N" yes and "H" no |
| **Postnatal check 2 Newborn** | Free text (ticked if provided) | No column |
| **OUTCOMES** |  |  |
| **Date and Time of Delivery** | Free text | Free text |
| **Type of delivery** | DGHS: Normal; Forceps/vacuum/breech; Caesarean; Same for DGFP, plus also tick option for Destructive operation. | Write Normal vaginal birth (KW); vacuum (VM); caesarean (CS); Beech (BR); other means (NY). |
| **Reason for CS** | DGFP only: Free text | **No column** |
| **GA (weeks)*** | Free text | **No column** |
| **Length of labour** | **No column** | Write "1" within 12 hours "2" after 12 hours |
| **Complications during admission (maternal)** | Write a description of any maternal complications during admission' | Write "AP" Vaginal bleeding, "PROM" Premature Rupture of Membranes, "A" Anaemia, "PE" Preeclampsia, "E" Eclampsia, Sepsis, Malaria, HIV+, FGM, etc. (using English language abbreviation) |
|  |  | Problems arose during and/or after childbirth, write: "PPH" Postpartum haemorrhage, "PE" Preeclampsia, "E" Eclampsia, "OL" Obstructed labour, 3rd-degree tear, "RP" Retained placenta, "MK" Chest pain, "KN" Fatigue, etc. (Mixed use of language) |
| **Female genital mutilation (FGM)** | **No column** | Write Yes "N" or No "H." |
| **Birth outcome (live or stillborn)** | Free text boxes for: live birth, fresh stillbirth, macerated stillbirth (ticked as relevant) (using English language abbreviation) | Write FSB (Fresh Still Birth) / MSB (Macerated Still Birth) (using English language abbreviation) |
| **Sex** | Tick: male, female, other | Gender of the child, write: female (KE), male (ME) |
| **Birth weight** | Write in grams (English instruction) | Write gram/kg (English instruction) |
| **APGAR score (1 and 5 minutes)** | Free text (APGAR at 10 mins written) | Free text |
| **Outcome at discharge (maternal)** | Write time and date of maternal discharge or death | Write H = Alive, A = Deceased with time and date of discharge (or death) |
| **Causes of Death (maternal)** | Write cause of maternal death | Write: ''Abortion"; "Sepsis"; ''Anaemia"; "PPH''; "OL for obstructed labour"; "PE for pre- Eclampsia''; as epilepsy of pregnancy; "RU" rupture of the uterus etc. |
| **Outcome at discharge (Baby)** | Write time and date of neonatal discharge or death | Write H = Alive, A = Deceased with time and date of discharge (or death) |
| **Causes of Death (Baby)** | Write cause of neonatal death | Write: 'Asphyxia' if it is failure to breathe; 'Sepsis' if it is infection; 'Prematurity' if born before week 37; 'Hypothermia' if it is lack of body temperature. 'Unknown' if the cause is unknown." |
| **Referral** | Write date and reason if newborn referred. | Write: i. Name of facility where mother originated. ii. Name of facility where mother referred to, e.g., for higher -level of care. iii. Write reason for the referral, (e.g., additional treatment for the child)." |

Emergency Obstetric Care (EmONC) Register, (Bangladesh)

Mfumo wa Taarifa za Uendeshaji wa Huduma za Afya (MTUHA), 12 (Tanzania)

*Preterm= less than 37 completed weeks, term =37 completed weeks or more
